# Supplementary material for: eSexualHealth: Preferences to use technology to promote sexual health among men who have sex with men and trans and gender diverse people
Source: Front Public Health. 2023 Jan 12;10:1064408. doi: 10.3389/fpubh.2022.1064408 (PMC9877518; doi:10.3389/fpubh.2022.1064408)
Supplement: Supplementary file 1 [file Data_Sheet_1.docx]

Supplementary Material

**Supplementary Table 1.** Preference for eHealth intervention platform, by risk group

| **Preferred Platform** | **GBMSM (%)** | **TGD (%)** | **Total** |
| --- | --- | --- | --- |
| Websites | 138 (32.2) | 12 (36.4) | 150 |
| Apps | 114 (26.6) | 5 (15.2) | 119 |
| Both website and apps | 145 (33.8) | 15 (45.5) | 160 |
| I wouldn't use either | 32 (7.5) | 1 (3.0) | 33 |

GBMSM, Gay and bisexual men who have sex with men; TGD, trans and gender diverse, p=0.228


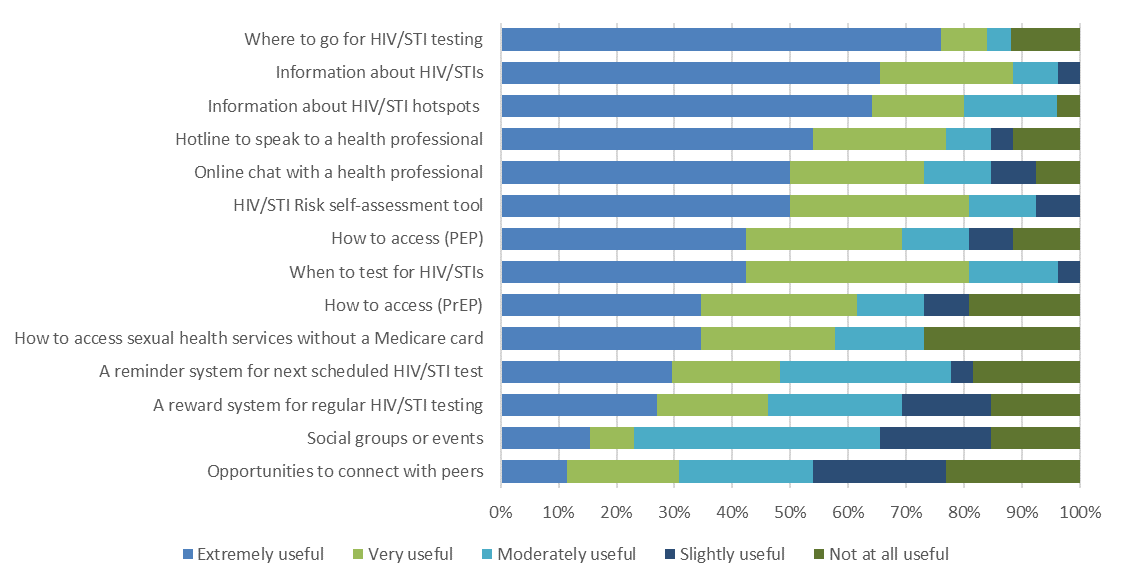


Supplementary Figure 2: Preferences for functions on a web-based platform among TGD people


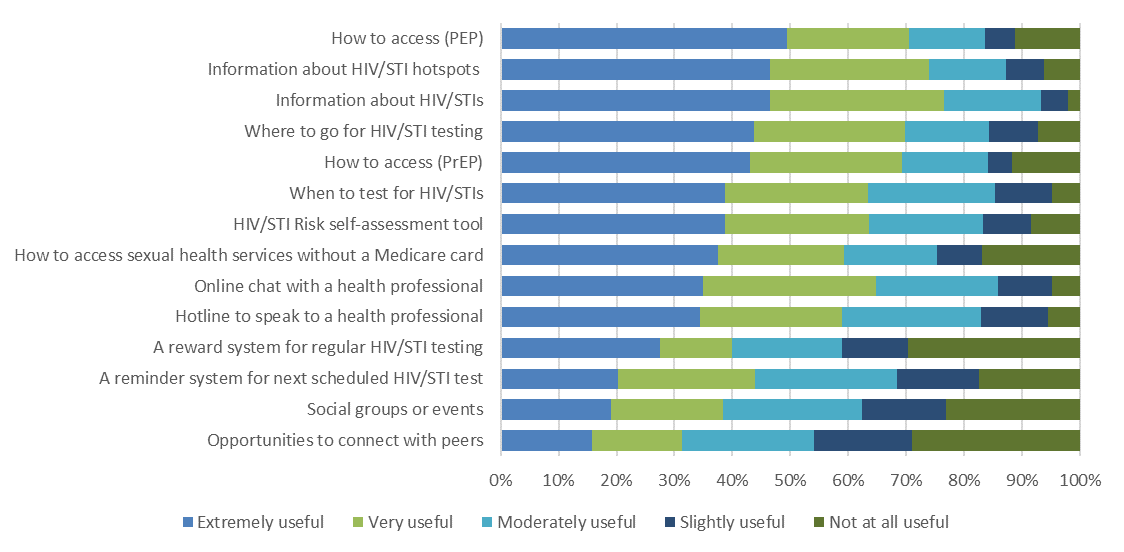


Supplementary Figure 1: Preferences for functions on a web-based platform among GBMSM


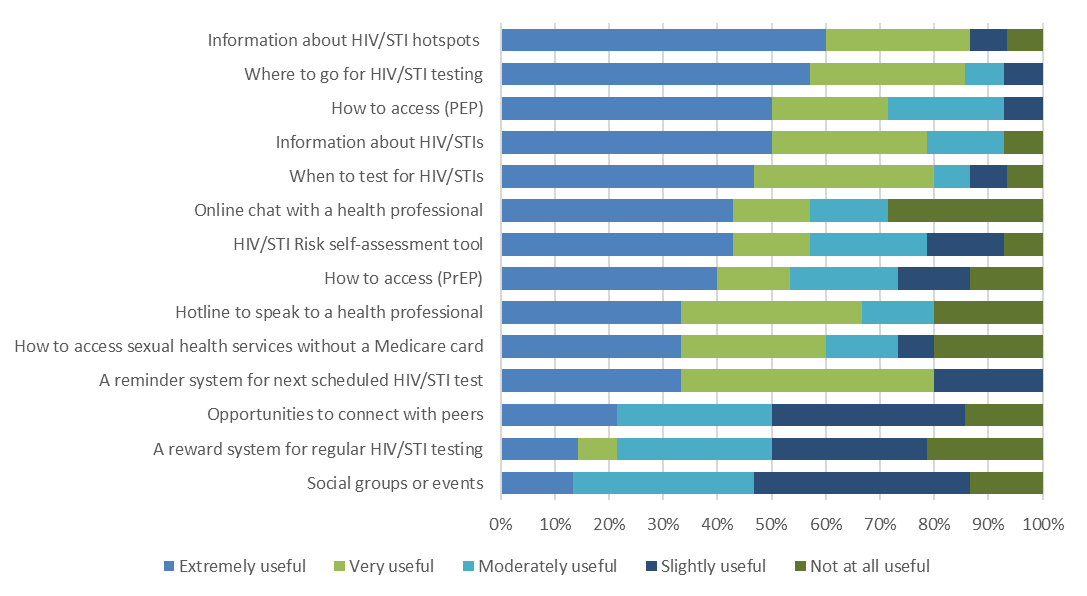


Supplementary Figure 4: Preferences for functions on an app-based platform among TGD people


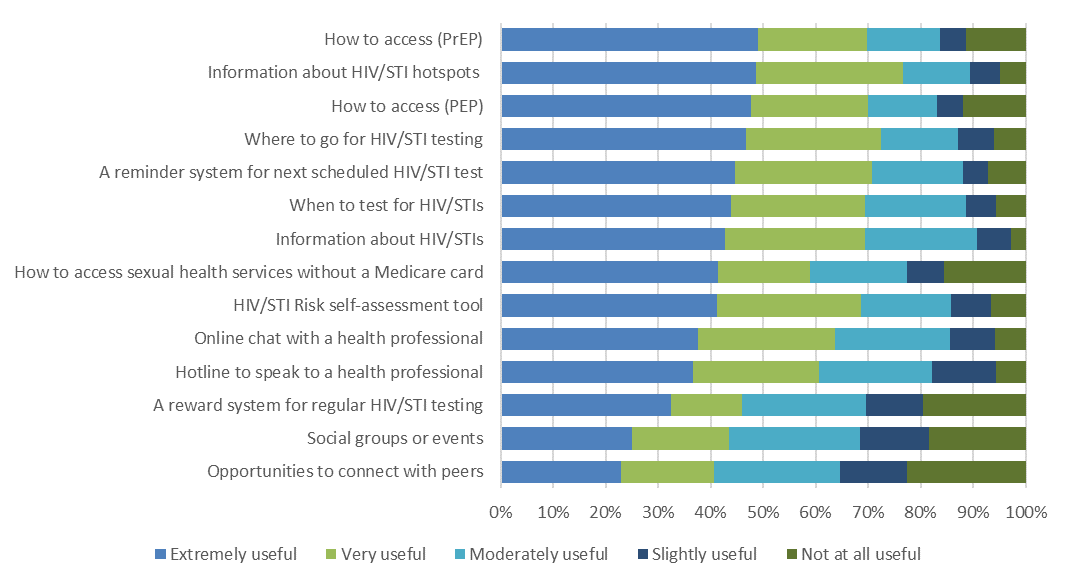


Supplementary Figure 3: Preferences for functions on an app-based platform among GBMSM


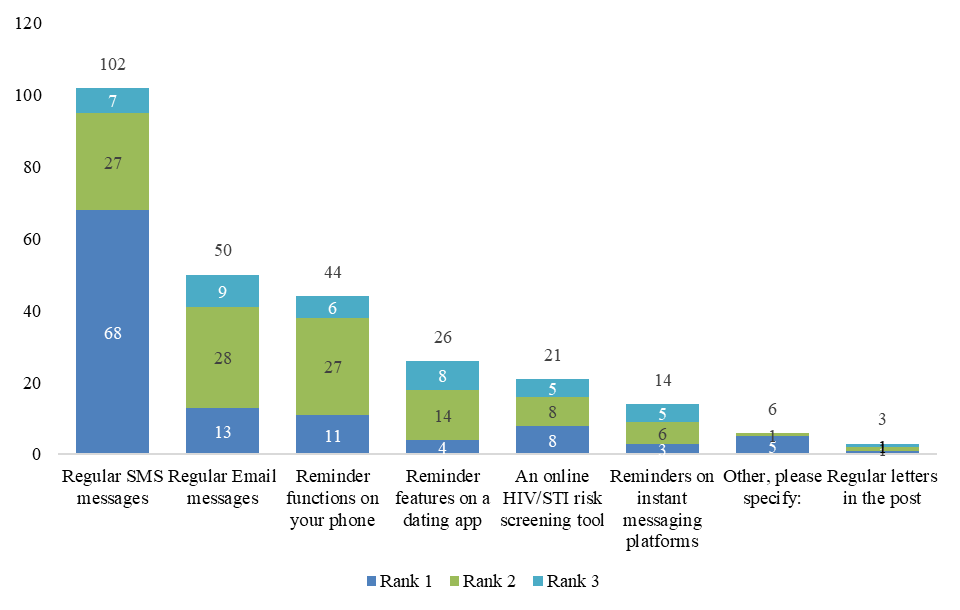


Supplementary Figure 5: Preferred HIV/STI testing reminder system on an eHealth platform among GBMSM


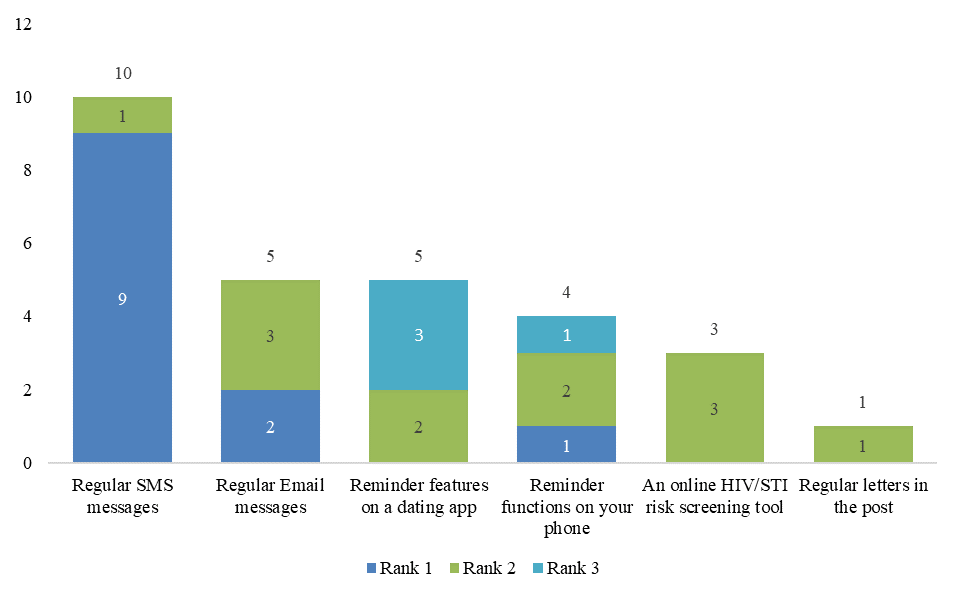


Supplementary Figure 6: Preferred HIV/STI testing reminder system on an eHealth platform, among TGD people
